# 2-(3-Bromo-4-ethyl­phen­yl)-2-methyl­propanoic acid

**DOI:** 10.1107/S1600536809036769

**Published:** 2009-09-16

**Authors:** Rong Sun, Xiao-Yan Yang, Cheng Yao

**Affiliations:** a Department of Applied Chemistry, College of Science, Nanjing University of Technology, Nanjing 210009, People’s Republic of China; b Department of Chemical Engineering, Nanjing College of Chemical Technology, Nanjing 210048, People’s Republic of China

## Abstract

In the title compound, C_12_H_15_BrO_2_, the carboxyl group forms a dihedral angle of 78.4 (3)° with the benzene ring plane. In the crystal, mol­ecules are linked into centrosymmetric dimers by pairs of O—H⋯O hydrogen bonds.

## Related literature

For the preparation of pharmaceuticals and active agrochemical ingredients using 2-(3-bromo-4-ethyl­phen­yl)-2-methyl­propanoic acid, see: Wiegand *et al.* (2007 ▶). For bond-length data, see: Allen *et al.* (1987 ▶).
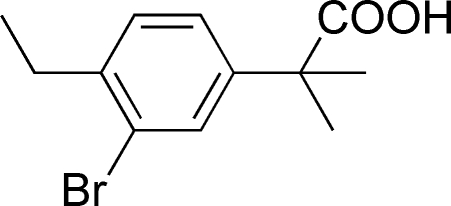


## Experimental

### 

#### Crystal data

C_12_H_15_BrO_2_
*M* *_r_* = 271.15
Monoclinic, 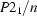

*a* = 9.7370 (19) Å
*b* = 7.2930 (15) Å
*c* = 17.433 (4) Å
β = 90.98 (3)°
*V* = 1237.8 (4) Å^3^
*Z* = 4
Mo *K*α radiation
μ = 3.30 mm^−1^
*T* = 298 K
0.20 × 0.10 × 0.10 mm

#### Data collection

Enraf–Nonius CAD-4 diffractometer
Absorption correction: ψ scan (North *et al.*, 1968 ▶) *T*_min_ = 0.558, *T*_max_ = 0.734
2389 measured reflections
2246 independent reflections
1171 reflections with *I* > 2σ(*I*)
*R*_int_ = 0.051
3 standard reflections every 200 reflections intensity decay: 1%

#### Refinement

*R*[*F* ^2^ > 2σ(*F* ^2^)] = 0.065
*wR*(*F* ^2^) = 0.157
*S* = 1.00
2246 reflections
136 parameters
H-atom parameters constrained
Δρ_max_ = 0.37 e Å^−3^
Δρ_min_ = −0.56 e Å^−3^

### 

Data collection: *CAD-4 Software* (Enraf–Nonius, 1985 ▶); cell refinement: *CAD-4 Software*; data reduction: *XCAD4* (Harms & Wocadlo, 1995 ▶); program(s) used to solve structure: *SHELXS97* (Sheldrick, 2008 ▶); program(s) used to refine structure: *SHELXL97* (Sheldrick, 2008 ▶); molecular graphics: *SHELXTL* (Sheldrick, 2008 ▶); software used to prepare material for publication: *SHELXTL*.

## Supplementary Material

Crystal structure: contains datablocks I, ls. DOI: 10.1107/S1600536809036769/ci2903sup1.cif

Structure factors: contains datablocks I. DOI: 10.1107/S1600536809036769/ci2903Isup2.hkl

Additional supplementary materials:  crystallographic information; 3D view; checkCIF report

## Figures and Tables

**Table 1:** Hydrogen-bond geometry (Å, °)

*D*—H⋯*A*	*D*—H	H⋯*A*	*D*⋯*A*	*D*—H⋯*A*
O1—H1*D*⋯O2^i^	0.82	1.88	2.696 (6)	178

Symmetry code: (i) 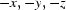.

**Fig. 1.**
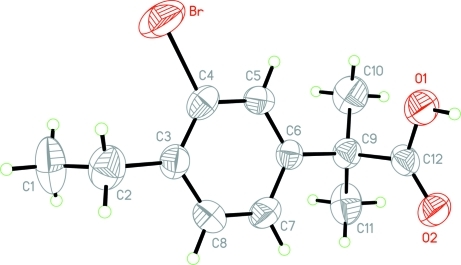
The molecular structure of the title compound, with the atom-numbering scheme. Displacement ellipsoids are drawn at the 50% probability level.

**Fig. 2.**
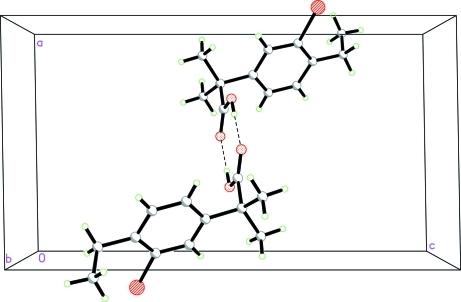
A packing diagram of the title compound. Hydrogen bonds are shown as dashed lines.

## supplementary crystallographic information

### Comment

2-(3-Bromo-4-ethylphenyl)-2-methylpropanoic acid is one of the valuable intermediates for the preparation of pharmaceuticals and active agrochemical ingredients (Wiegand *et al.*, 2007). We report here the crystal structure of the title compound.

Bond lengths (Allen *et al.*, 1987) and angles in the title molecule (Fig.1) are within normal ranges. The plane of the carboxyl group forms a dihedral angle of 78.4 (3)° with the benzene plane.

In the crystal, molecules are linked into centrosymmetric dimers by pairs of O—H···O hydrogen bonds (Fig. 2).

### Experimental

The title compound was prepared by the hydrolyzation of methyl 2-(3-bromo-4-ethylphenyl)-2-methylpropanoate (10.42 g, 0.037 mol) in a solution of methanol (30 ml) and acetone (150 ml), catalyzed by KOH aqueous solution (73 ml, 1.0 mol/l) at room temperature (298 k). After stirring for 8 h, methanol and acetone were removed by reduced distillation to obtain an aqueous substrate. The substrate was washed with dichloromethane (4× 20 ml), and precipitated with concentrated hydrochloric acid. Then the precipitate was washed with water, collected and dried to give 2-(3-bromo-4-ethylphenyl)-2-methylpropanoic acid (4.07 g, 0.015 mol) with a yield of 41.0%. Single crystals of the compound were obtained by slow evaporation of an methanol solution at room temperature.

### Refinement

H atoms were positioned geometrically, with O-H = 0.82 Å and C-H = 0.93 and 0.96 Å for aromatic and methyl H, respectively, and constrained to ride on their parent atoms, with *U*_iso_(H) = *xU*_eq_(C,O), where *x* = 1.2 for aromatic H and *x* = 1.5 for other H atoms.

### Crystal data

**Table:**

C_12_H_15_BrO_2_	*F*(000) = 552
*M**_r_* = 271.15	*D*_x_ = 1.455 Mg m^−^^3^
Monoclinic, *P*2_1_/*n*	Mo *K*α radiation, λ = 0.71073 Å
Hall symbol: -P 2yn	Cell parameters from 25 reflections
*a* = 9.7370 (19) Å	θ = 10–13°
*b* = 7.2930 (15) Å	µ = 3.30 mm^−^^1^
*c* = 17.433 (4) Å	*T* = 298 K
β = 90.98 (3)°	Block, colourless
*V* = 1237.8 (4) Å^3^	0.20 × 0.10 × 0.10 mm
*Z* = 4	

### Data collection

**Table:**

Enraf–Nonius CAD-4 diffractometer	1171 reflections with *I* > 2σ(*I*)
Radiation source: fine-focus sealed tube	*R*_int_ = 0.051
graphite	θ_max_ = 25.3°, θ_min_ = 2.4°
ω/2θ scans	*h* = 0→11
Absorption correction: ψ scan (North *et al.*, 1968)	*k* = 0→8
*T*_min_ = 0.558, *T*_max_ = 0.734	*l* = −20→20
2389 measured reflections	3 standard reflections every 200 reflections
2246 independent reflections	intensity decay: 1%

### Refinement

**Table:**

Refinement on *F*^2^	Primary atom site location: structure-invariant direct methods
Least-squares matrix: full	Secondary atom site location: difference Fourier map
*R*[*F*^2^ > 2σ(*F*^2^)] = 0.065	Hydrogen site location: inferred from neighbouring sites
*wR*(*F*^2^) = 0.157	H-atom parameters constrained
*S* = 1.00	*w* = 1/[σ^2^(*F*_o_^2^) + (0.073*P*)^2^] where *P* = (*F*_o_^2^ + 2*F*_c_^2^)/3
2246 reflections	(Δ/σ)_max_ = 0.001
136 parameters	Δρ_max_ = 0.37 e Å^−^^3^
0 restraints	Δρ_min_ = −0.56 e Å^−^^3^

### Special details

**Table:**

Geometry. All e.s.d.'s (except the e.s.d. in the dihedral angle between two l.s. planes) are estimated using the full covariance matrix. The cell e.s.d.'s are taken into account individually in the estimation of e.s.d.'s in distances, angles and torsion angles; correlations between e.s.d.'s in cell parameters are only used when they are defined by crystal symmetry. An approximate (isotropic) treatment of cell e.s.d.'s is used for estimating e.s.d.'s involving l.s. planes.
Refinement. Refinement of *F*^2^ against ALL reflections. The weighted *R*-factor *wR* and goodness of fit *S* are based on *F*^2^, conventional *R*-factors *R* are based on *F*, with *F* set to zero for negative *F*^2^. The threshold expression of *F*^2^ > σ(*F*^2^) is used only for calculating *R*-factors(gt) *etc*. and is not relevant to the choice of reflections for refinement. *R*-factors based on *F*^2^ are statistically about twice as large as those based on *F*, and *R*- factors based on ALL data will be even larger.

### Fractional atomic coordinates and isotropic or equivalent isotropic displacement parameters (Å^2^)

**Table:**

	*x*	*y*	*z*	*U*_iso_*/*U*_eq_	
Br	0.59774 (8)	0.20831 (13)	0.22133 (4)	0.0837 (4)	
O1	0.1885 (4)	0.0103 (6)	0.0039 (3)	0.0702 (14)	
H1D	0.1216	−0.0561	0.0099	0.105*	
C1	0.5163 (8)	0.6662 (12)	0.2979 (4)	0.095 (3)	
H1A	0.5349	0.6986	0.3504	0.142*	
H1B	0.4845	0.7723	0.2703	0.142*	
H1C	0.5988	0.6212	0.2751	0.142*	
O2	0.0282 (4)	0.2132 (6)	−0.0248 (3)	0.0621 (12)	
C2	0.4083 (6)	0.5204 (10)	0.2946 (3)	0.0651 (19)	
H2A	0.3259	0.5656	0.3186	0.078*	
H2B	0.4402	0.4145	0.3234	0.078*	
C3	0.3738 (6)	0.4625 (9)	0.2136 (3)	0.0456 (15)	
C4	0.4468 (5)	0.3321 (9)	0.1736 (3)	0.0453 (15)	
C5	0.4157 (5)	0.2833 (8)	0.0980 (3)	0.0420 (14)	
H5A	0.4688	0.1960	0.0733	0.050*	
C6	0.3068 (5)	0.3641 (8)	0.0596 (3)	0.0376 (14)	
C7	0.2310 (6)	0.4946 (9)	0.0993 (3)	0.0551 (17)	
H7A	0.1569	0.5521	0.0751	0.066*	
C8	0.2650 (6)	0.5387 (9)	0.1738 (4)	0.0585 (18)	
H8A	0.2115	0.6251	0.1987	0.070*	
C9	0.2652 (6)	0.3128 (8)	−0.0232 (3)	0.0458 (15)	
C10	0.3853 (7)	0.2252 (10)	−0.0659 (4)	0.069 (2)	
H10A	0.3562	0.1951	−0.1173	0.103*	
H10B	0.4141	0.1157	−0.0397	0.103*	
H10C	0.4605	0.3102	−0.0675	0.103*	
C11	0.2130 (7)	0.4781 (9)	−0.0680 (4)	0.067 (2)	
H11A	0.1368	0.5316	−0.0419	0.100*	
H11B	0.1840	0.4401	−0.1185	0.100*	
H11C	0.2853	0.5669	−0.0720	0.100*	
C12	0.1475 (6)	0.1738 (8)	−0.0153 (3)	0.0456 (15)	

### Atomic displacement parameters (Å^2^)

**Table:**

	*U*^11^	*U*^22^	*U*^33^	*U*^12^	*U*^13^	*U*^23^
Br	0.0699 (5)	0.1100 (7)	0.0703 (5)	0.0306 (5)	−0.0219 (4)	0.0089 (5)
O1	0.055 (3)	0.041 (3)	0.113 (4)	−0.007 (2)	−0.016 (3)	0.011 (3)
C1	0.103 (6)	0.102 (7)	0.078 (5)	−0.038 (6)	−0.012 (5)	−0.032 (5)
O2	0.046 (3)	0.046 (3)	0.094 (3)	−0.001 (2)	−0.016 (2)	0.007 (2)
C2	0.063 (4)	0.082 (5)	0.050 (4)	−0.007 (4)	0.000 (3)	−0.003 (4)
C3	0.042 (3)	0.053 (4)	0.041 (3)	−0.003 (3)	−0.002 (3)	−0.005 (3)
C4	0.035 (3)	0.054 (4)	0.047 (3)	−0.007 (3)	−0.002 (3)	0.010 (3)
C5	0.041 (3)	0.040 (3)	0.045 (3)	0.004 (3)	0.002 (3)	0.000 (3)
C6	0.038 (3)	0.036 (3)	0.039 (3)	−0.005 (3)	0.001 (3)	0.001 (3)
C7	0.046 (4)	0.065 (5)	0.054 (4)	0.011 (3)	−0.013 (3)	−0.006 (4)
C8	0.059 (4)	0.054 (5)	0.062 (4)	0.009 (3)	−0.003 (3)	−0.019 (3)
C9	0.048 (3)	0.048 (4)	0.041 (3)	0.000 (3)	−0.005 (3)	−0.001 (3)
C10	0.067 (4)	0.090 (6)	0.049 (4)	0.000 (4)	0.008 (3)	−0.009 (4)
C11	0.089 (5)	0.064 (5)	0.047 (4)	−0.014 (4)	−0.017 (4)	0.013 (4)
C12	0.057 (4)	0.034 (4)	0.045 (3)	0.001 (3)	−0.011 (3)	−0.001 (3)

### Geometric parameters (Å, °)

**Table:**

Br—C4	1.904 (6)	C5—H5A	0.93
O1—C12	1.299 (7)	C6—C7	1.395 (8)
O1—H1D	0.82	C6—C9	1.539 (7)
C1—C2	1.496 (9)	C7—C8	1.373 (8)
C1—H1A	0.96	C7—H7A	0.93
C1—H1B	0.96	C8—H8A	0.93
C1—H1C	0.96	C9—C11	1.520 (8)
O2—C12	1.205 (7)	C9—C10	1.536 (9)
C2—C3	1.505 (8)	C9—C12	1.538 (8)
C2—H2A	0.97	C10—H10A	0.96
C2—H2B	0.97	C10—H10B	0.96
C3—C8	1.374 (8)	C10—H10C	0.96
C3—C4	1.383 (8)	C11—H11A	0.96
C4—C5	1.394 (8)	C11—H11B	0.96
C5—C6	1.376 (7)	C11—H11C	0.96
			
C12—O1—H1D	109.5	C8—C7—H7A	119.7
C2—C1—H1A	109.5	C6—C7—H7A	119.7
C2—C1—H1B	109.5	C7—C8—C3	123.7 (6)
H1A—C1—H1B	109.5	C7—C8—H8A	118.2
C2—C1—H1C	109.5	C3—C8—H8A	118.2
H1A—C1—H1C	109.5	C11—C9—C10	109.3 (5)
H1B—C1—H1C	109.5	C11—C9—C12	109.0 (5)
C1—C2—C3	112.4 (6)	C10—C9—C12	110.1 (5)
C1—C2—H2A	109.1	C11—C9—C6	111.7 (5)
C3—C2—H2A	109.1	C10—C9—C6	111.5 (5)
C1—C2—H2B	109.1	C12—C9—C6	105.1 (4)
C3—C2—H2B	109.1	C9—C10—H10A	109.5
H2A—C2—H2B	107.8	C9—C10—H10B	109.5
C8—C3—C4	115.0 (5)	H10A—C10—H10B	109.5
C8—C3—C2	121.2 (6)	C9—C10—H10C	109.5
C4—C3—C2	123.8 (5)	H10A—C10—H10C	109.5
C3—C4—C5	123.2 (5)	H10B—C10—H10C	109.5
C3—C4—Br	120.3 (4)	C9—C11—H11A	109.5
C5—C4—Br	116.5 (5)	C9—C11—H11B	109.5
C6—C5—C4	120.2 (5)	H11A—C11—H11B	109.5
C6—C5—H5A	119.9	C9—C11—H11C	109.5
C4—C5—H5A	119.9	H11A—C11—H11C	109.5
C5—C6—C7	117.5 (5)	H11B—C11—H11C	109.5
C5—C6—C9	122.6 (5)	O2—C12—O1	123.0 (6)
C7—C6—C9	119.9 (5)	O2—C12—C9	123.3 (5)
C8—C7—C6	120.5 (6)	O1—C12—C9	113.7 (5)
			
C1—C2—C3—C8	−95.0 (8)	C2—C3—C8—C7	178.3 (6)
C1—C2—C3—C4	84.7 (8)	C5—C6—C9—C11	−144.6 (6)
C8—C3—C4—C5	1.5 (9)	C7—C6—C9—C11	36.7 (7)
C2—C3—C4—C5	−178.3 (6)	C5—C6—C9—C10	−22.0 (8)
C8—C3—C4—Br	−178.4 (4)	C7—C6—C9—C10	159.4 (6)
C2—C3—C4—Br	1.9 (8)	C5—C6—C9—C12	97.3 (6)
C3—C4—C5—C6	−0.9 (9)	C7—C6—C9—C12	−81.4 (6)
Br—C4—C5—C6	178.9 (4)	C11—C9—C12—O2	−18.2 (8)
C4—C5—C6—C7	0.2 (8)	C10—C9—C12—O2	−138.1 (6)
C4—C5—C6—C9	−178.5 (5)	C6—C9—C12—O2	101.7 (6)
C5—C6—C7—C8	−0.2 (9)	C11—C9—C12—O1	163.1 (5)
C9—C6—C7—C8	178.5 (6)	C10—C9—C12—O1	43.2 (7)
C6—C7—C8—C3	0.9 (11)	C6—C9—C12—O1	−77.0 (6)
C4—C3—C8—C7	−1.5 (10)		

### Hydrogen-bond geometry (Å, °)

**Table:**

*D*—H···*A*	*D*—H	H···*A*	*D*···*A*	*D*—H···*A*
O1—H1D···O2^i^	0.82	1.88	2.696 (6)	178

Symmetry codes: (i) −*x*, −*y*, −*z*.
